# Structural and functional brain network alterations in prenatal alcohol exposed neonates

**DOI:** 10.1007/s11682-020-00277-8

**Published:** 2020-04-18

**Authors:** Annerine Roos, Jean-Paul Fouche, Jonathan C. Ipser, Katherine L. Narr, Roger P. Woods, Heather J. Zar, Dan J. Stein, Kirsten A. Donald

**Affiliations:** 1 SU/UCT MRC Unit on Risk and Resilience in Mental Disorders, Department of Psychiatry, Stellenbosch University, PO Box 241, 8000 Cape Town, South Africa; 2 Department of Paediatrics and Child Health, University of Cape Town, Cape Town, South Africa; 3 Department of Psychiatry and Mental Health, University of Cape Town, Cape Town, South Africa; 4 Departments of Neurology and of Psychiatry and Biobehavioral Sciences, University of California, Los Angeles, USA

**Keywords:** prenatal alcohol exposure, neonate, multimodal brain imaging, structural brain network, functional brain network, graph theoretical analysis

## Abstract

Prenatal alcohol exposure leads to alterations in cognition, behavior and underlying brain architecture. However, prior studies have not integrated structural and functional imaging data in children with prenatal alcohol exposure. The aim of this study was to characterize disruptions in both structural and functional brain network organization after prenatal alcohol exposure in very early life. A group of 11 neonates with prenatal alcohol exposure and 14 unexposed controls were investigated using diffusion weighted structural and resting state functional magnetic resonance imaging. Covariance networks were created using graph theoretical analyses for each data set, controlling for age and sex. Group differences in global hub arrangement and regional connectivity were determined using nonparametric permutation tests. Neonates with prenatal alcohol exposure and controls exhibited similar global structural network organization. However, global functional networks of neonates with prenatal alcohol exposure comprised of temporal and limbic hubs, while hubs were more distributed in controls representing an early default mode network. On a regional level, controls showed prominent structural and functional connectivity in parietal and occipital regions. Neonates with prenatal alcohol exposure showed regionally, predominant structural and functional connectivity in several subcortical regions and occipital regions. The findings suggest early functional disruption on a global and regional level after prenatal alcohol exposure and indicate suboptimal organization of functional networks. These differences likely underlie sensory dysregulation and behavioral difficulties in prenatal alcohol exposure.

## Introduction

The early period of brain development represents a critical time during which effects of prenatal exposures may be embedded and have impact for life. The brain develops rapidly during the first year of life and is fundamentally connected by two years of age, while functional specialization continues throughout childhood into adulthood (Gao et al. 2017). However, core regional networks are discernable even in the neonatal brain. The infant brain typically has short neural connections that are strongest in primary sensorimotor and visual cortices (Fransson et al. 2011; Keunen et al. 2017). Subcortical projections, including with the thalamus, support regional integration of these networks (Alcauter et al. 2014; Smyser et al. 2015; Toulmin et al. 2015). Connections between medial frontal and parietal association regions are also present, but are yet to become fully integrated parts of the default-mode network (DMN) (Gao et al. 2017). Primary networks adapt over time to favor longer-range connections that balance the cost of segregation and integration of brain networks (Cao et al. 2017; Vertes and Bullmore 2015).

Structural and functional networks develop in tandem. Structural networks provide the inherent physical framework that facilitates and limits functional network development and integration, thus both networks should ideally be included when studying potential insults to development (Grayson and Fair 2017). Structural hubs or highly connected regions in the neonate are similar to those observed in the adult brain, except that connections are refined with age, to improve strength and effectiveness, while functional network hubs gradually move from primary to higher order association regions to support greater functional specialization (Keunen et al. 2017).

Alterations in cognition, behavior and underlying brain architecture due to prenatal alcohol exposure (PAE) have been well-documented in children older than five years (Donald et al. 2015; Lebel et al. 2012). Recent advances in infant imaging techniques have given impetus to investigations of normal brain development during the first two years of life, and to data on brain network connectivity (Graham et al. 2015a; Vertes and Bullmore 2015). Few studies, however, have addressed the effects that PAE may have on early brain network organization. Such work is crucial given that PAE may lead to impairments in self-regulation, adaptive behavior, and cognition in young children (Garrison et al. 2019).

To our knowledge, only one study has investigated brain network connectivity in neonates exposed to alcohol prenatally. The study found functional connectivity disruptions in neonates using resting state MRI between sensorimotor and striatal networks that underlie motor function (Donald et al. 2016a). Similarly, functional MRI studies in older children and adolescents continue to demonstrate disruptions in sensorimotor network connectivity after PAE (e.g. Long et al. 2018). Further, a study using structural MRI, found decreased subcortical gray matter volume in neonates following PAE (Donald et al. 2016b), while another study found a smaller corpus callosum after heavy PAE (Jacobson et al. 2017) that has implications for network integration. The aim of this study was to apply graph theoretical analysis to multimodal MRI data to investigate “small-world” networks of the brain in neonates (Hosseini et al. 2012), allowing the simultaneous characterization of disruptions in structural and functional brain network organization after PAE. We expect that regions (or nodes) of core structural and functional networks underlying primary sensorimotor functions will be altered on a global and regional level in the PAE group compared to controls. We also posit that these alterations will be most notable on a regional level given prominent local neurodevelopmental patterns in the neonate.

## Methods

This investigation is a sub study of the Drakenstein Child Health Study (DCHS) that is following mother-child dyads from mid-pregnancy until children are at least five years of age. Detailed methodology for the core DCHS study is described elsewhere (Stein et al. 2015). Maternal substance use was assessed using the World Health Organization’s Alcohol, Smoking and Substance Involvement Screening Test (ASSIST) with a cutoff score of 11 for moderate to high risk of problem drinking (Humeniuk et al. 2008; Jackson et al. 2010). Mothers without a positive urine screen for any illicit drugs (Lozano et al. 2007); with neonates who were not premature (i.e. > = 36 weeks); did not have a history of hypoxic ischemic encephalopathy, genetic syndrome or other obvious congenital abnormalities or other evidence of neonatal medical complications; were approached for participation in the study. Mothers provided written informed consent for participation in the main and sub study.

Ethical approval for the main study was obtained from the Human Research Ethics Committee of the Faculty of Health Sciences, University of Cape Town (UCT REF 401/2009) and this sub study was independently reviewed and approved (UCT REF 525/2012). The study was conducted according to the guidelines of the 1964 Helsinki Declaration.

### Brain imaging

At the brain imaging visit, 2–4 week old neonates with PAE and control neonates without any substance exposure history were imaged during natural sleep without sedation. Groups had similar sex and age distributions (Table 1). Structural diffusion tensor imaging and functional resting state scans were acquired using a 3T Siemens Allegra MRI scanner. T2-weighted structural images had the following parameters: TR 3500 ms; TE 354 ms; 128 slices; slice thickness 1 mm; voxel size 1.0 × 1.0 × 1.0 mm. Diffusion images had the following parameters: 45 non-collinear gradient directions; TR 7900 ms; TE 90 ms; slice thickness 1.6 mm; b-values 0 and 1000s/mm^2^; voxel size 1.3 × 1.3 × 1.6 mm^3^. Resting state gradient echo T2-weighted echo planar images (EPI) were acquired with the following parameters: TR 2000 ms; TE 30 ms; flip angle = 77°; 33 slices; slice thickness 4 mm; voxel resolution = 2.5 × 2.5 × 4.0 mm.

**Table 1:** Demographic and anthropometric information of neonates

	PAE	Controls	Statistics
Age in days (SD)	21.9 (4.3)	23.7 (6.1)	F = 0.81, p = 0.39
Gestation in weeks (SD)	39.5 (2.3)	39.1 (1.5)	F = 0.29, p = 0.60
Boys/girls (n)	5/9	6/5	χ^2^ = 0.89, p = 0.35
Length (cm)	51.6	51.6	F = 0.001, p = 0.98
Weight (kg)	4.2	4.0	F = 0.63, p = 0.44
Head circumference (cm)	36.3	36.5	F = 0.08, p = 0.79

PAE, prenatal alcohol exposed

Diffusion data was acquired in 50 neonates and resting state data in 36 neonates. After excluding 2 premature neonates and images failing quality control (see next section), the final sample were: PAE = 11 and controls = 14. For both the diffusion and resting state data analysis, the University of California (UNC) neonate structural template was used for registration purposes (Shi et al. 2011). Data processing steps are described in detail in Table 2.

**Table 2:** Data processing steps

Processing	Program	Steps and descriptions
Diffusion tensor imaging	TORTOISE	1. Axialization of images (similar to MIPAV) to optimize alignment without warping or changing intensity parameters, by calculating an affine alignment to the UNC neonate structural template.
		2. DIFFPREP: Distortion corrections for participant motion, eddy currents and basic echo-planar imaging (EPI) distortions separately on each anterior-posterior and posterior-anterior encoded image.
		3. DR-BUDDI: Merging encoded sets and further EPI distortion corrections.
	AFNI	4. Post processing and diffusion tensor parameter fitting.
Resting state	AFNI	1. Stabilizing magnetic field by removing first four EPI volumes per scan.
		2. Exclusion of outlier signal intensities per voxel using 3dDespike.
		3. Motion correction by rigid-body alignment of each EPI to the third volume, and resampling of data to 2.5 mm in three spatial dimensions.
		4. Intermediate anatomical registration to T2 images to derive displacement factors and final registration to UNC neonate atlas.
		5. Spatial smoothing using 5 mm full width at half-maximum (FWHM).
		6. Registration of individual resting state images to UNC neonate atlas, and utilization of the 90 regions as masks to extract time series data.
Graph theoretical analysis	GAT	1. Creation of small-world networks.
		2. Threshholding of association matrices at a range of network densities.
		3. Extraction of clustering coefficient that provides an indication of local segregation of networks i.e. mean connectivity among nodes.
		3. Extraction of characteristic path length that provides an indication of network integration i.e. mean shortest path length between nodes.
		4. Creation of random networks with regions and edges comparable to that of the actual brain network, to evaluate the clustering coefficient and characteristic path length, and determine network arrangement.
	BCT	5. Estimation of nodal betweenness centrality that determines all shortest path lengths of connections between local regions.
		6. Identification of hubs based on nodal betweenness centrality output.
		6. Nonparametric permutation testing (1000 permutations) to investigate group differences. Comparison of small-world index, clustering coefficient and characteristic path length by group across a range of densities (0.1 to 0.4); and regional network measures e.g. nodal betweenness centrality at minimum density (0.1).

AFNI, Analysis of Functional NeuroImages; BCT, Brain Connectivity Toolbox; GAT, Graph Theoretical Analysis Toolbox; MIPAV, Medical Image Processing, Analysis, and Visualization; TORTOISE, Tolerably Obsessive Registration and Tensor Optimization Indolent Software Ensemble; UNC, University of California

### Diffusion data processing

Diffusion data of each participant were visually inspected for motion and signal dropout retaining a minimum of 14 volumes for further processing. See Table 3 for detail on volumes discarded by group. TORTOISE v2.5.2 (Irfanoglu et al. 2014; Pierpaoli et al. 2010; Wu et al. 2008) was used for image preprocessing due to comprehensive correction and anatomical registration relevant to pediatric data (Taylor et al. 2016) (Table 2). AFNI was used for diffusion tensor parameter fitting (Cox 1996).

**Table 3:** Outcome of quality checking procedures for diffusion and resting state data. For diffusion data, the number of volumes discarded on average (out of 48) due to motion or other technical issues, and group differences are shown. For resting state data, the number of volumes discarded (out of 176) due to excessive motion, and the maximum and average motion (mm) over all volumes before and after removal of volumes are shown, as well as group differences. Although more volumes were removed and average motion was higher in the prenatal alcohol exposed group, this was not significantly different compared to controls.

	PAE		Controls		
	mean	SE	mean	SE	Statistics
Diffusion data					
Volumes removed	15	3.06	10	2.18	F = 1.67, p = 0.21
Resting state data					
Volumes removed	20	4.72	17	3.04	F = 0.47, p = 0.50
Maximum motion before	3.61	0.41	4.27	0.65	F = 0.65, p = 0.43
Maximum motion after	2.58	0.32	4.10	0.65	F = 3.76, p = 0.06
Average motion before	0.14	0.02	0.12	0.01	F = 1.49, p = 0.23
Average motion after	0.08	0.01	0.07	0.01	F = 1.68, p = 0.21

The TrackVis toolkit (Wang et al. 2007) was used to perform deterministic tractography on diffusion weighted images. Fractional anisotropy, fiber numbers and volume were calculated for each region for structural brain network creation.

### Resting state data processing

Resting state data were preprocessed using AFNI (Table 2). Images with motion artifact were removed i.e. points that were displaced by > = 0.3 mm relative to an earlier time point (Table 3). Regions defined by registration to the UNC neonate atlas were utilized as masks to extract time series data from the resting state images for functional brain network creation.

### Graph theoretical analysis

For diffusion structural networks, the UNC neonate track regions for which FA was calculated were incorporated as nodes. Edges were determined to be any connection with a fiber number of > 3 between regions (Rubinov and Sporns 2010). The fiber number was multiplied by the FA value and divided by the regional volume to account for differences in region size. From this data, an association matrix was created.

For the resting state functional networks, nodes were defined based on the average BOLD intensity within voxels constituting each UNC atlas region for each volume within the time series (Rubinov and Sporns 2010). The edges were defined as the Pearson correlations between the mean time series for pairs of regions, and an association matrix was constructed.

In separate graph theoretical analyses, small-world networks were created using the Graph Theoretical Analysis Toolbox (Hosseini et al. 2012) (Table 2). Diffusion data and resting state association matrices were threshholded at a range of network densities; at a minimum density of 0.1 where networks were not fragmented, to a maximum density of 0.4 where connections are still deemed biological (He et al. 2008; Hosseini et al. 2013; Kaiser and Hilgetag 2006). Key defining network parameters of the global network included the small-worldness, clustering coefficient and the characteristic path length (Hosseini et al. 2013; Rubinov and Sporns 2010).

The Brain Connectivity Toolbox (Rubinov and Sporns 2010) was used to quantify the global and regional network measures. Parameters included the global network parameters; and global and local efficiency; nodal betweenness centrality, modularity, assortativity and transitivity. P-values for each of the group results were FDR-corrected. Regional network connectivity was estimated as nodal betweenness centrality at minimum density (Hosseini et al. 2013). These findings subsequently provide information on regions that have highest network influence globally i.e. hubs were regions with connectivity 2 SD above that of mean network connectivity (Bernhardt et al. 2011). Hub regions are key in coordinating the flow of information in the brain and globally most connected. Differences between groups in brain network organization were determined using nonparametric permutation tests that control for multiple comparisons (see Nichols and Hayasaka (2003) for detail).

## Results

### Structural connectivity

**Fig. 1:**
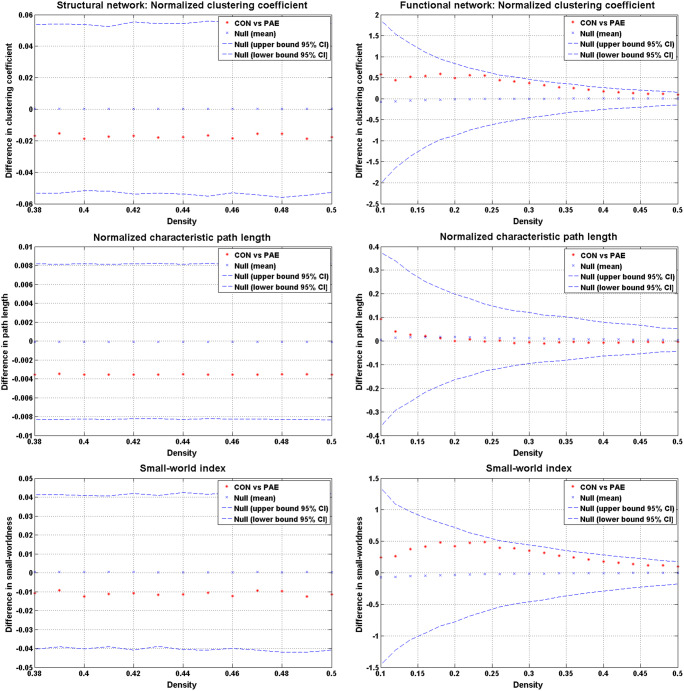
Between-group differences and 95% confidence intervals in normalized global network measures as a function of network density. There were no significant differences in any of the parameters between groups, thus no ‘x’ fell outside of the confidence intervals. CON, controls; PAE, prenatal alcohol exposed

The structural network had a minimum density of full network connectivity of 0.38 that defines a small-world network found within the brain (Hosseini et al. 2012). Parameters defining the global network across densities did not differ between groups (Fig. 1). The normalized clustering coefficient was > 1 (p = 0.55), the normalized characteristic path length was close to 1 (p = 0.43), and the small-world index was > 1 (p = 0.61) as expected (Bassett and Bullmore 2006).

Concerning regional connectivity, there were differences in nodal betweenness centrality of regions. PAE neonates had significantly higher connectivity in temporal, occipital and frontal regions compared to controls, while controls had higher connectivity in parietal regions (Table 4).

**Table 4:** Group differences in node betweenness centrality of structural and functional networks as an indication of regional connectivity

	PAE > Controls				Controls > PAE			
Lobe	Structural	p	Functional	p	Structural	p	Functional	p
Temporal	R AMYG	0.044	R AMYG	0.013				
	L STP	0.013	L STP	0.029				
			R STP	0.013				
	R FG	0.038						
Occipital	L SOG	0.003					R LNG	0.043
Frontal	L MFG	0.007	R IFG-T	0.022				
Parietal					R MCG	0.023	R PCUN	0.017
					R ROL	0.046		

PAE, prenatal alcohol exposed; AMYG, amygdala; FG, fusiform gyrus; IFG-T, inferior frontal gyrus (triangularis); LNG, lingual gyrus; MCG, middle cingulate gyrus; MFG, middle frontal gyrus; PCUN, precuneus; ROL, rolandic operculum; SOG, superior occipital gyrus; STP, superior temporal pole

**Fig. 2:**
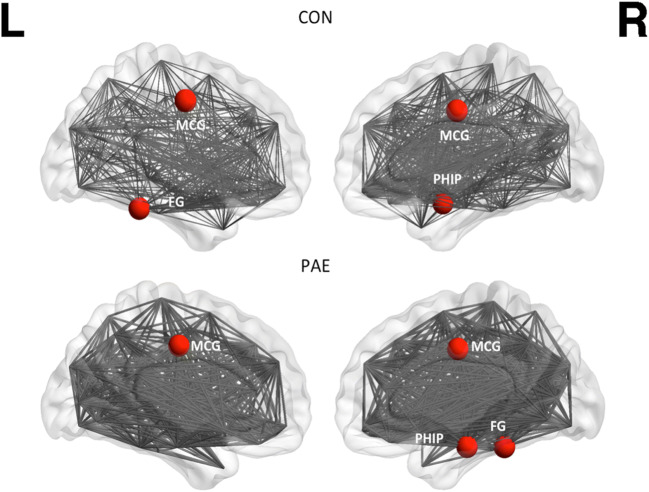
Hubs of the structural network of the control (CON) and PAE group as derived from diffusion tensor imaging data. These were regions with connectivity 2 SD above that of mean network connectivity. Hubs indicated by circles are the middle cingulate gyrus (MCG), fusiform gyrus (FG) and parahippocampal gyrus (PHIP)

Concerning global connectivity, PAE and control neonates had similar hubs in their structural networks including parietal and temporal regions (Table 5, Fig. 2). There were no group differences in global or local efficiency as markers of network resilience, or other network parameters (e.g. assortativity, modularity, transitivity).

**Table 5:** Highly connected network hubs

	PAE	Controls
Structural hubs	Bilateral MCG	Bilateral MCG
	R PHIP	R PHIP
	R FG	L FG
Functional hubs	R AMYG	R CALC
	R PLD	L PCG
	R STP	R LNG
		R CUN
		R PCUN

PAE, prenatal alcohol exposed; AMYG, amygdala; CALC, calcarine; CUN, cuneus; FG; fusiform gyrus; LNG, lingual gyrus; MCG, middle cingulate gyrus; PLD, pallidum; PCUN, precuneus; PCG, posterior cingulate gyrus; PHIP, parahippocampal gyrus; STP, superior temporal pole

### Functional connectivity

The minimum density of full network connectivity was 0.10. The constructed networks followed a small-world organization across densities that was not different between groups. Networks were balanced between segregation and integration (Fig. 1): the normalized clustering coefficient was > 1 (p = 0.27), the normalized characteristic path length was close to 1 (p = 0.79), and the small-world index was > 1 (p = 0.29).

Concerning regional connectivity, there were differences in nodal betweenness centrality of regions (Table 4). PAE neonates had significantly higher connectivity in temporal and frontal regions compared to controls, while controls had greater connectivity in parietal and occipital regions.

**Fig. 3:**
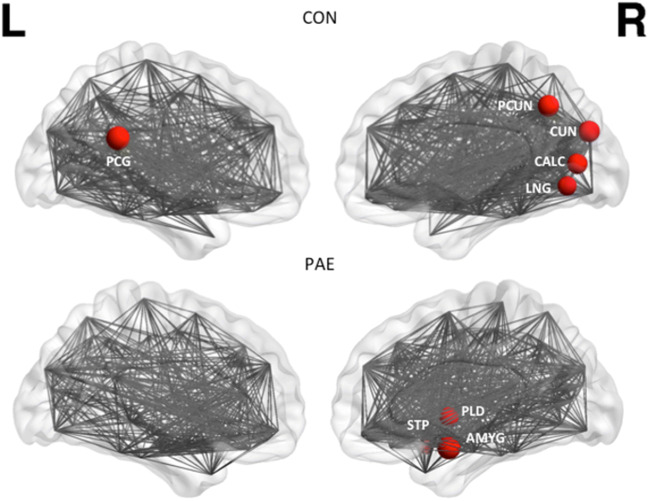
Hubs of the functional network of the control (CON) and PAE group as derived from resting state data. These were regions with connectivity 2 SD above that of mean network connectivity. Hubs indicated by circles are the posterior cingulate gyrus (PCG), superior temporal pole (STP), pallidum (PLD), amygdala (AMYG), precuneus (PCUN), cuneus (CUN), calcarine sulcus (CALC) and lingual gyrus (LNG)

Concerning global connectivity, hubs in PAE neonates were dominant in subcortical regions (basal ganglia and temporal and limbic regions), while hubs in controls were dominant in parietal and occipital regions (Table 5, Fig. 3). There were no group differences in global or local efficiency of networks or other network parameters.

## Discussion

This study compared structural and functional brain network connectivity after PAE with that in unexposed neonates at a time when core brain networks are being established. Structural and functional networks in both PAE and unexposed neonates adhered to small-world network properties, indicative of the brain and commonalities in networks. We observed pronounced group differences in functional network connectivity of global and regional networks in neonates with PAE compared to those without alcohol exposure during pregnancy. These differences likely reflect disrupted prenatal neural programming due to alcohol toxicity.

### Early global connectivity

Structural network hubs were similar in both neonates with PAE and controls consistent with work indicating stable structural compared to functional networks in healthy neonates (Keunen et al. 2017; Vertes and Bullmore 2015). Hubs are key as they maintain network resilience (Rubinov and Sporns 2010). Early structural networks provide an inherent physical framework that shapes functional network development (Cao et al. 2017; Huang et al. 2015). Specifically, the neonatal brain has regional prominence to support primary sensory and motor functions, thus is somewhat segregated before becoming integrated.

In functional networks of unexposed neonates, key regions of the emerging DMN were present including the precuneus, posterior cingulate gyrus and cuneus, consistent with previous work (Gao et al. 2017; Vertes and Bullmore 2015). These regions become functionally efficient on a global level with time. The calcarine hub also integrates to support DMN network function with age, while temporal regions that were functional hubs in the group with PAE but not the control group, should decrease in regional efficiency with development (Huang et al. 2015) to favor global efficiency. The presence of base-level temporal and limbic functional hubs in PAE, compared to control neonates in whom more distributed hubs in posterior cortical regions were observed (suggesting initial integration of higher order networks), may indicate early signs of disrupted and suboptimal organization of functional networks in PAE. Previous work in infancy following exposures to alcohol and/or other substances support our findings in neonates with PAE of altered connectivity of the amygdala and pallidum in functional networks (Donald et al. 2016a; Scott-Goodwin et al. 2016). Subcortical regions support early integration of cortical connectivity (Gao et al. 2017). However, these discrete hubs demonstrated in our group with PAE may not integrate effectively with atypically connected key cortical regions in later development.

### Prominent regional connectivity

In unexposed neonates, our regional findings confirm that primary localized structural connectivity is in place in parietal and occipital regions that underlie basic sensorimotor, visual, perceptive and auditory function (Alcauter et al. 2014; Fransson et al. 2009). Functional resting state studies have demonstrated involvement of the precuneus and lingual gyrus in visual attention in adults (Goldin et al. 2008), while the precuneus has also been implicated in speech perception in infants (Dehaene-Lambertz et al. 2002); these are expected emerging functions in infancy. The middle cingulate gyrus that showed higher structural connectivity in unexposed neonates compared to those with PAE may contribute to the trajectory of sensorimotor development in time (Vogt 2016).

In contrast, in neonates with PAE, findings implicated relatively diffuse extra and multiple regions outside the parietal lobe with higher regional connectivity compared to controls. Excessive local connections may hinder sufficient shifting of regions towards global integration (Huang et al. 2015). These findings after PAE suggest atypical connectivity patterns that may hinder primary and higher-order functional specialization with time.

The regional structural and functional connectivity patterns in the PAE group showed some overlap, validating the use of this multimodal approach to elicit a better understanding of microstructure in relation to developing functional connections (Skudlarski et al. 2008; Smyser et al. 2015). This overlap may suggest that the local structural foundation to primary functional specialization is altered by PAE, and in particular, relating to the right amygdala and superior temporal pole, and regions of the frontal gyrus. Alcohol affect neural plasticity of the amygdala, temporal regions and cortex (see Scott-Goodwin et al. 2016 for mechanisms). We previously reported significant differences in structural volumes, including higher bilateral amygdala volume in neonates with PAE compared to controls (Donald et al. 2016b). Other functional MRI studies investigating the early effects of prenatal substance exposure in 2 to 6 week old infants, including alcohol, found hyper-connectivity of the amygdala with frontal regions (Grewen et al. 2015; Salzwedel et al. 2015). Amygdala-frontal circuitry has been implicated in regulation of arousal, while self-regulation has been shown to be affected in infants with PAE (Garrison et al. 2019). Higher structural connectivity in the middle frontal gyrus in this cohort following PAE may indicate higher arousal as networks mature. The effect of postnatal environments may further impact this trajectory. Six-month-old infants with higher negative emotionality living in a volatile environment had greater connectivity of middle prefrontal cortex regions with the posterior cingulate gyrus of the DMN (Graham et al. 2015b).

The higher connectivity found in the superior temporal pole and inferior frontal gyrus in neonates with PAE compared to unexposed neonates may represent networks that process faces in infancy. Facial processing involves attention, auditory and language domains in order to make sense of sensory cues and assist language development (Dehaene-Lambertz et al. 2002; Paterson et al. 2006; Tzourio-Mazoyer et al. 2002). This early network interaction typically develops to later involve the fusiform gyrus as part of higher-order perceptual processing networks (Paterson et al., 2006), while after PAE this gyrus already had atypically higher connectivity. Considering that connectivity in the fusiform gyrus, and frontal lobe is not as pronounced in healthy developing neonates (Geng et al. 2012), this may indicate heightened vigilance to sensory cues after PAE. Indeed, consistent with our findings in temporal and frontal regions, altered functional connectivity was found using resting state MRI between facial sensorimotor regions and the insula and frontal regions in PAE children suggesting sensory processing difficulties (Long et al. 2018). Children with fetal alcohol spectrum disorders showed difficulty in recognizing facial emotional stimuli, which has implications for social-emotional development (Kerns et al. 2016). Interestingly, the temporal lobe was implicated in youth with a familial history of alcohol use who demonstrated emotional face processing deficits (Cservenka 2016). Further, higher connectivity of the superior occipital gyrus after PAE is likely associated with heightened somatosensory function given anatomical overlap with dorsal attention and DMN networks (Gao et al. 2013).

### Comparing structural and functional networks

Networks derived by diffusion tensor imaging and resting state data show high overlap due to both representing a slow time scale (Batista-Garcia-Ramo and Fernandez-Verdecia 2018). However, functional networks do not account for all anatomical connections as functional correlations may result from indirect connections and dynamic fluctuations (Vertes and Bullmore 2015). Differences between these networks are also attributed to genetics (Cao et al. 2017), and in how the respective correlation networks are created (Hosseini and Kesler 2013).

Regarding this cohort, structural and functional hubs were different, similar to Cao et al. (2017), who attributed these patterns to different modes of maturation that converge in young childhood. Instead, consistent with previous work, there was prominent overlapping regional connectivity in neonates (Smyser et al. 2011). The neonatal period up to two years is characterized by considerable myelination and pruning of neurons. Since the relatively stable structural network confines functional development while allowing for dynamic configuration (Cao et al. 2017), key regions are expected to integrate gradually from primary to higher-order networks, thus strengthening the global structure-function relationship (Fransson et al. 2011; Vertes and Bullmore 2015).

## Limitations and strengths

Several limitations and strengths should be emphasized. First, although sample size was small the age-window was narrow. Our sample size was comparable to other studies that have used either diffusion tensor imaging or resting state modalities separately in neonates with prenatal substance exposure (e.g. Grewen et al. 2015; Taylor et al. 2015). Second, this was a cross-sectional study. Ideally, studies should assess structural and functional network development in a longitudinal study design in order to better assess the clinical significance and causality of early alterations. Nevertheless, our findings are consistent with much of what is known about normal brain development in neonates as well as neural alterations following prenatal substance exposure. The study provides important clues on early interrelatedness of co-developing structural and functional networks following PAE.

## Conclusion

PAE disrupts the global and regional architecture of brain networks and this is already evident during the first month of life. Although the same structural hubs were present in both groups, key functional regions that should lead integration of the DMN were not present as hubs in PAE; consistent with aberrant connectivity that has implications for future strength and functionality of core networks. Longitudinal studies are required to determine whether lower connectivity of key hubs implicate delays, disruptions or compensatory responses to PAE in childhood. There were clear differences in structural-functional network arrangements after PAE, suggesting suboptimal connectivity. Larger apparent group differences in functional compared to structural brain organization suggests that structural differences may only emerge as the brain matures and as functional demand increases. Atypical regional connectivity that was found in temporal, frontal and occipital regions likely underlie sensory dysregulation and hypervigilance (e.g. self-regulation difficulties), and other behavioral difficulties in infants with PAE.

## References

[CR1] Alcauter S, Lin W, Smith XJK, Short SJ, Goldman BD, Reznick JS (2014). Development of thalamocortical connectivity during infancy and its cognitive correlations. Journal of Neuroscience.

[CR2] Batista-Garcia-Ramo, K., & Fernandez-Verdecia, C. I. (2018). What we know about the brain structure-function relationship. Behavioral Sciences 8(4). 10.3390/bs8040039. 10.3390/bs8040039 PMC5946098 29670045

[CR3] Bassett, D. S., & Bullmore, E. (2006). Small-world brain networks. *Neuroscientist*, *12*(6), 512–523. https://doi.org/12/6/512. 10.1177/1073858406293182 17079517

[CR4] Bernhardt BC, Chen Z, He Y, Evans AC, Bernasconi N (2011). Graph-theoretical analysis reveals disrupted small-world organization of cortical thickness correlation networks in temporal lobe epilepsy. Cerebral Cortex.

[CR5] Cao M, Huang H, He Y (2017). Developmental connectomics from infancy through early childhood. Trends in Neurosciences.

[CR6] Cox RW (1996). AFNI: software for analysis and visualization of functional magnetic resonance neuroimages. Computers and Biomedical Research.

[CR7] Cservenka A (2016). Neurobiological phenotypes associated with a family history of alcoholism. Drug and Alcohol Dependence.

[CR8] Dehaene-Lambertz G, Dehaene S, Hertz-Pannier L (2002). Functional neuroimaging of speech perception in infants. Science.

[CR9] Donald KA, Eastman E, Howells FM, Adnams C, Riley EP, Woods RP (2015). Neuroimaging effects of prenatal alcohol exposure on the developing human brain: a magnetic resonance imaging review. Acta Neuropsychiatrica.

[CR10] Donald KA, Fouche JP, Roos A, Koen N, Howells FM, Riley EP (2016). Alcohol exposure in utero is associated with decreased gray matter volume in neonates. Metabolic Brain Disease.

[CR11] Donald KA, Ipser JC, Howells FM, Roos A, Fouche JP, Riley EP (2016). Interhemispheric functional brain connectivity in neonates with prenatal alcohol exposure: preliminary findings. Alcoholism: Clinical and Experimental Research.

[CR12] Fransson P, Aden U, Blennow M, Lagercrantz H (2011). The functional architecture of the infant brain as revealed by resting-state fMRI. Cerebral Cortex.

[CR13] Fransson P, Skiold B, Engstrom M, Hallberg B, Mosskin M, Aden U (2009). Spontaneous brain activity in the newborn brain during natural sleep: an fMRI study in infants born at full term. Pediatric Research.

[CR14] Gao W, Gilmore JH, Shen D, Smith JK, Zhu H, Lin W (2013). The Synchronization within and interaction between the default and dorsal attention networks in early infancy. Cerebral Cortex.

[CR15] Gao, W., Lin, W., Grewen, K., & Gilmore, J. H. (2017). Functional connectivity of the infant human brain: plastic and modifiable. *The Neuroscientist : a Review Journal Bringing Neurobiology, Neurology and Psychiatry*, *23*(2), 169–184. 10.1177/1073858416635986 10.1177/1073858416635986 PMC5145769 26929236

[CR16] Garrison L, Morley S, Chambers CD, Bakhireva LN (2019). Forty years of assessing neurodevelopmental and behavioral effects of prenatal alcohol exposure in infants: what have we learned?. Alcoholism, Clinical and Experimental Research.

[CR17] Geng X, Gouttard S, Sharma A, Gu H, Styner M, Lin W (2012). Quantitative tract-based white matter development from birth to age 2 years. NeuroImage.

[CR18] Goldin PR, McRae K, Ramel W, Gross JJ (2008). The neural bases of emotion regulation: reappraisal and suppression of negative emotion. Biological Psychiatry.

[CR19] Graham AM, Pfeifer JH, Fisher PA, Carpenter S, Fair DA (2015). Early life stress is associated with default system integrity and emotionality during infancy. Journal of Child Psychology and Psychiatry, and Allied Disciplines.

[CR20] Graham AM, Pfeifer JH, Fisher PA, Lin W, Gao W, Fair DA (2015). The potential of infant fMRI research and the study of early life stress as a promising exemplar. Developmental Cognitive Neuroscience.

[CR21] Grayson DS, Fair DA (2017). Development of large-scale functional networks from birth to adulthood: A guide to the neuroimaging literature. NeuroImage.

[CR22] Grewen K, Salzwedel AP, Gao W (2015). Functional connectivity disruption in neonates with prenatal marijuana exposure. Frontiers in Human Neuroscience.

[CR23] He Y, Chen Z, Evans A (2008). Structural insights into aberrant topological patterns of large-scale cortical networks in Alzheimer’s disease. The Journal of Neuroscience.

[CR24] Hosseini SM, Black JM, Soriano T, Bugescu N, Martinez R, Raman MM (2013). Topological properties of large-scale structural brain networks in children with familial risk for reading difficulties. NeuroImage.

[CR25] Hosseini SM, Hoeft F, Kesler SR (2012). GAT: a graph-theoretical analysis toolbox for analyzing between-group differences in large-scale structural and functional brain networks. PloS One.

[CR26] Hosseini, S. . M. ., & Kesler, S. . R. . (2013). Comparing connectivity pattern and small-world organization between structural correlation and resting-state networks in healthyadults.. *Neuroimage*, *78*, 402–414. 10.1016/j.neuroimage.2013.04.032 10.1016/j.neuroimage.2013.04.032 PMC3673883 23603348

[CR27] Huang H, Shu N, Mishra V, Jeon T, Chalak L, Wang ZJ, Rollins N (2015). Development of human brain structural networks through infancy and childhood. Cerebral Cortex.

[CR28] Humeniuk R, Ali R, Babor TF, Farrell M, Formigoni ML, Jittiwutikarn J (2008). Validation of the Alcohol, Smoking And Substance Involvement Screening Test (ASSIST). Addiction.

[CR29] Irfanoglu MO, Modi P, Nayak A, Knutsen A, Sarlls J, Pierpaoli C (2014). DR-BUDDI: diffeomorphic registration for blip up-down diffusion imaging. Medical Image Computing and Computer-Assisted Intervention.

[CR30] Jackson PB, Williams DR, Stein DJ, Herman A, Williams SL, Redmond DL (2010). Race and psychological distress: the South African Stress and Health study. Journal of Health and Social Behavior.

[CR31] Jacobson SW, Jacobson JL, Molteno CD, Warton CMR, Wintermark P, Hoyme HE (2017). Heavy prenatal alcohol exposure is related to smaller corpus callosum in newborn MRI scans. Alcoholism, Clinical and Experimental Research.

[CR32] Kaiser, M., & Hilgetag, C. C. (2006). Nonoptimal component placement, but short processing paths, due to long-distance projections in neural systems. *PLoS Computational Biology*, *2*(7), e95. https://doi.org/06-PLCB-RA-0069R3. 10.1371/journal.pcbi.0020095 PMC1513269 16848638

[CR33] Kerns KA, Siklos S, Baker L, Muller U (2016). Emotion recognition in children with Fetal Alcohol Spectrum Disorders. Child Neuropsychology.

[CR34] Keunen K, Counsell SJ, Benders MJNL (2017). The emergence of functional architecture during early brain development. NeuroImage.

[CR35] Lebel C, Mattson SN, Riley EP, Jones KL, Adnams CM, May PA (2012). A longitudinal study of the long-term consequences of drinking during pregnancy: heavy in utero alcohol exposure disrupts the normal processes of brain development. Journal of Neuroscience.

[CR36] Long X, Little G, Beaulieu C, Lebel C (2018). Sensorimotor network alterations in children and youth with prenatal alcohol exposure. Human Brain Mapping.

[CR37] Lozano J, Garcia-Algar O, Vall O, de la Torre R, Scaravelli G, Pichini S (2007). Biological matrices for the evaluation of in utero exposure to drugs of abuse. Therapeutic Drug Monitoring.

[CR38] Nichols, T. E., & Hayasaka, S. (2003). Controlling the familywise error rate in functional neuroimaging: a comparative review. *Statistical Methods in Medical Research*, *12(5)*, 419–446. 10.1191/0962280203sm341ra 14599004

[CR40] Paterson SJ, Heim S, Friedman JT, Choudhury N, Benasich AA (2006). Development of structure and function in the infant brain: implications for cognition, language and social behaviour. Neuroscience and Biobehavioral Reviews.

[CR41] Pierpaoli, C., Walker, L., Irfanoglu, M. O., Barnett, A., Basser, P., Chang, L. C., et al. (2010). TORTOISE: An integrated software package for processing of diffusion MRI data. In *ISMRM (2010) 18th Annual Meeting* (p. 1597). Stockholm, Sweden.

[CR42] Rubinov M, Sporns O (2010). Complex network measures of brain connectivity: uses and interpretations. NeuroImage.

[CR43] Salzwedel AP, Grewen XKM, Vachet XC, Gerig G, Lin W, Gao XW (2015). Prenatal drug exposure affects neonatal brain functional connectivity. Journal of Neuroscience.

[CR44] Scott-Goodwin AC, Puerto M, Moreno I (2016). Toxic effects of prenatal exposure to alcohol, tobacco and other drugs. Reproductive Toxicology.

[CR45] Shi F, Yap PT, Wu G, Jia H, Gilmore JH, Lin W, Shen D (2011). Infant brain atlases from neonates to 1- and 2-year-olds. PloS One.

[CR46] Skudlarski P, Jagannathan K, Calhoun VD, Hampson M, Skudlarska BA, Pearlson G (2008). Measuring brain connectivity: diffusion tensor imaging validates resting state temporal correlations. NeuroImage.

[CR47] Smyser CD, Neil JJ, Louis S (2015). Use of resting-state functional MRI to study brain development and injury in neonates. Seminars in Perinatology.

[CR48] Smyser CD, Snyder AZ, Neil JJ (2011). Functional connectivity MRI in infants: exploration of the functional organization of the developing brain. Neuroimage.

[CR49] Stein DJ, Koen N, Donald KA, Adnams CM, Koopowitz S, Lund C (2015). Investigating the psychosocial determinants of child health in Africa: The Drakenstein Child Health Study. Journal of Neuroscience Methods.

[CR50] Taylor PA, Alhamud A, van der Kouwe A, Saleh MG, Laughton B, Meintjes E (2016). Assessing the performance of different DTI motion correction strategies in the presence of EPI distortion correction. Human Brain Mapping.

[CR51] Taylor PA, Jacobson SW, van der Kouwe A, Molteno CD, Chen G, Wintermark P (2015). A DTI-based tractography study of effects on brain structure associated with prenatal alcohol exposure in newborns. Human Brain Mapping.

[CR52] Toulmin H, Beckmann CF, O’Muircheartaigh J, Ball G, Nongena P, Makropoulos A (2015). Specialization and integration of functional thalamocortical connectivity in the human infant. Proceedings of the National Academy of Sciences of the United States of America.

[CR53] Tzourio-Mazoyer N, De Schonen S, Crivello F, Reutter B, Aujard Y, Mazoyer B (2002). Neural correlates of woman face processing by 2-month-old infants. NeuroImage.

[CR54] Vertes PE, Bullmore ET (2015). Annual research review: growth connectomics – the organization and reorganization of brain networks during normal and abnormal development. Journal of Child Psychology and Psychiatry, and Allied Disciplines.

[CR55] Vogt BA (2016). Midcingulate cortex: structure, connections, homologies, functions and diseases. Journal of Chemical Neuroanatomy.

[CR56] Wang R, Benner T, Sorensen AG, Wedeen VJ (2007). Diffusion toolkit: a software package for diffusion imaging data processing and tractography. Proceedings of the International Society for Magnetic Resonance in Medicine.

[CR57] Wu M, Chang LC, Walker L, Lemaitre H, Barnett AS, Marenco S, Pierpaoli C (2008). Comparison of EPI distortion correction methods in diffusion tensor MRI using a novel framework. Medical Image Computing and Computer-Assisted Intervention.

